# Localized Surface Plasmon Resonance Decorated with Carbon Quantum Dots and Triangular Ag Nanoparticles for Chlorophyll Detection

**DOI:** 10.3390/nano12010035

**Published:** 2021-12-23

**Authors:** Nur Afifah Ahmad Nazri, Nur Hidayah Azeman, Mohd Hafiz Abu Bakar, Nadhratun Naiim Mobarak, Yunhan Luo, Norhana Arsad, Tg Hasnan Tg Abd Aziz, Ahmad Rifqi Md Zain, Ahmad Ashrif A. Bakar

**Affiliations:** 1 Department of Electrical, Electronic and Systems Engineering, Faculty of Engineering and Built Environment, Universiti Kebangsaan Malaysia, Bangi 43600, Malaysia; p105424@siswa.ukm.edu.my (N.A.A.N.); hafiz.bujei95@gmail.com (M.H.A.B.); noa@ukm.edu.my (N.A.); 2 Department of Chemical Sciences, Faculty of Sciences and Technology, Universiti Kebangsaan Malaysia, Bangi 43600, Malaysia; nadhratunnaiim@ukm.edu.my; 3 Guangdong Provincial Key Laboratory of Optical Fiber Sensing and Communications, College of Science and Engineering, Jinan University, Guangzhou 510632, China; yunhanluo@163.com; 4 Institute of Microengineering and Nanoelectronics, Universiti Kebangsaan Malaysia, Bangi 43600, Malaysia; hasnanaziz@ukm.edu.my; 5 Institut Islam Hadhari, Universiti Kebangsaan Malaysia, Bangi 43600, Malaysia

**Keywords:** carbon quantum dots, localized surface plasmon resonance, silver nanoparticles, chlorophyll, optical sensor

## Abstract

This paper demonstrates carbon quantum dots (CQDs) with triangular silver nanoparticles (AgNPs) as the sensing materials of localized surface plasmon resonance (LSPR) sensors for chlorophyll detection. The CQDs and AgNPs were prepared by a one-step hydrothermal process and a direct chemical reduction process, respectively. FTIR analysis shows that a CQD consists of NH_2_, OH, and COOH functional groups. The appearance of C=O and NH_2_ at 399.5 eV and 529.6 eV in XPS analysis indicates that functional groups are available for adsorption sites for chlorophyll interaction. A AgNP–CQD composite was coated on the glass slide surface using (3-aminopropyl) triethoxysilane (APTES) as a coupling agent and acted as the active sensing layer for chlorophyll detection. In LSPR sensing, the linear response detection for AgNP–CQD demonstrates R^2^ = 0.9581 and a sensitivity of 0.80 nm ppm^−1^, with a detection limit of 4.71 ppm ranging from 0.2 to 10.0 ppm. Meanwhile, a AgNP shows a linear response of R^2^ = 0.1541 and a sensitivity of 0.25 nm ppm^−1^, with the detection limit of 52.76 ppm upon exposure to chlorophyll. Based on these results, the AgNP–CQD composite shows a better linearity response and a higher sensitivity than bare AgNPs when exposed to chlorophyll, highlighting the potential of AgNP–CQD as a sensing material in this study.

## 1. Introduction

Various types of nutrients exist in the oceans, such as chlorophyll, nitrate, and phosphate [1]. The excessive amount of nutrients and chlorophyll present in the sea surface area leads to algae biomass growth. It prevents the sunlight from reaching other aquatic plants, preventing them from photosynthesis. The nutrient-rich environment may also lead to eutrophication issues [2]. Chlorophyll has become an essential water quality estimator of phytoplankton biomass because it is specific to algae and is directly proportional to algae biomass even in the presence of non-algae inorganic and inorganic particles [3,4]. Therefore, several analytical techniques have been developed for the determination of chlorophyll, including high-performance liquid chromatography (HPLC) [5], fluorometry [6], and spectrophotometry [7]. Among them, fluorometry was considered an optimal method for chlorophyll analysis because it requires less sample, is sensitive and rapid, and can be used for in situ measurements [8]. However, these analytical techniques are unstable, laboratory based, and time consuming and detect chlorophyll concentrations with low accuracy [8]. As per another report, chlorophyll concentration and harmful phytoplankton biomass can also be detected using remote monitoring of ocean colors [9]. However, this technique is unable to detect small variations in chlorophyll concentration. Hence, it is significant to develop a sensitive and selective output method.

Localized surface plasmon resonance (LSPR) sensors are commonly used to measure the low chemical and biological analytes concentration. They are capable of detecting the target in real time, in a repeatable and susceptible manner [10,11]. The noble metal nanoparticles (NPs), such as gold (Au) and silver (Ag), possess unique optoelectrical properties due to prominent LSPR characteristics. The LSPR phenomenon’s absorption range is influenced by the surrounding medium’s shape, size, substances, and refractive index. For example, the triangular NPs of Ag have three different sharp “corners” or “tips,” which make significant contributions to their optical, chemical, electrical, and unique surface plasmon resonance properties [12]. The anisotropic morphology of the triangular NP will exhibit more LSPR bands and decrease the symmetry. In addition, compared with spherical or quasi-spherical AgNPs, the edge length can quickly destroy the in-plane dipole resonance [13]. Again, some theoretical models and experiments show that the AgNPs’ triangular tips provide significant local field enhancement and high spatial resolution because most of the signal is produced from the tip region [14]. In every sensor platform, the correlation coefficient is one of the critical parameters to determine sensor performance. A significant linear correlation coefficient can confirm a significant relationship between the dependent and independent variables. For example, the coefficient of determination R2 indicates how much precision the explained value of the dependent variable provides and how much accuracy for the independent variable [15]. Kim et al. state that local field enhancement can significantly improve LSPR detection with excellent correlation coefficients and high sensitivity over a wide dynamic range [16]. However, as related signs of progress, the requirements for measurement precision are increasing and the sensitivity of LSPR sensors must be enhanced further.

Nowadays, nanomaterials are widely used in various analytical methods, with the development of nanoscience and nanotechnology. Carbon quantum dots (CQDs), also known as carbon dots, have been commonly used in many applications due to their outstanding photoluminescent characteristics, ease of modifying and functionalizing, high stability, and good biocompatibility [17]. Due to their numerous surface functional groups, including hydroxyl, amino, and carboxyl, CQDs can act as sensing materials for detecting analytes based on their charge transfer or electrostatic interactions with analytes, confirmed by experimental studies and density functional theoretical (DFT) calculations [18,19,20]. Furthermore, the functional groups and good hydrophilicity properties on the surface of CQDs promote their use as support for designing optical sensors for the quantitative determination of metal ions, macromolecules, and environmental contaminants [21,22].

Monitoring chlorophyll levels in water bodies, such as seas and lakes, is a direct way to track algae growth. Excessive growth of algae in the water bodies leads to the eutrophication issue, which can endanger aquatic life and public health. Due to this reason, the development of an algae monitoring system is crucial to prevent such phenomena from occurring, thus ensuring the safety of water usage in the future. This research reports a CQD- and AgNP-based LSPR sensing platform for chlorophyll. The CQDs and triangular AgNPs were prepared via hydrothermal and direct chemical approaches at ambient temperature. The prepared layer’s structure, functional group, and morphology were analyzed using X-ray spectroscopy, Fourier-transform infrared spectroscopy (FTIR), field-emission scanning microscopy (FESEM), transmission electron microscopy (TEM), and fluorescence spectroscopy. The synthesized CQDs contained citric acid and ethylenediamine, providing a heteroatom to the core and surface functional group. The localized SPR sensor was carried out as the optical sensing technique using the proposed sensing material to detect chlorophyll, and a sensitivity of 0.80 nm ppm^−1^ with a limit of detection of 4.71 ppm was achieved. 

## 2. Materials and Methods

Research materials: Citric acid, silver nitrate, trisodium citrate, sodium borohydride, 3-aminopropyltrimethoxysilane (97%), ethanol, and acetone were purchased from Sigma Aldrich, Spruce Street, Saint Louis, MO, USA. Ethylenediamine was purchased from R&M Chemicals, Dundee, UK, and total chlorophyll was purchased from Tokyo Chemical Industry Co Ltd, Chuoku, Tokyo, Japan. Standard chlorophyll solutions were prepared by stepwise dilution of 1000 mg L^−1^ chlorophyll solution before use. Deionized water was used throughout the experiment.

**Figure 1:**
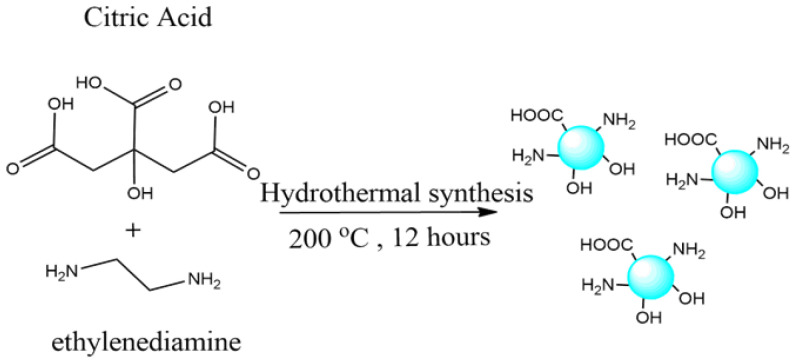
Synthesis of CQDs.

Synthesis of CQD: According to the published process, CQD was prepared by one step of a hydrothermal method with some modification (Figure 1) [23]. First, 1.0507 g of citric acid was dissolved in deionized water (10 mL). After that, 335 μL of ethylenediamine was added into the solution and transferred to a 100 mL Teflon-lined autoclave. The resulting solution was kept at 200 °C for 12 h. After being cooled at room temperature naturally, the CQD solution was centrifuged (4000 rpm, 20 min), filtered using a 0.22 µm membrane filter, and stored at 4 °C for further use.

Synthesis of AgNPs: The triangular silver nanoparticle solution was synthesized according to a reported method using a direct chemical reduction process at room temperature [13]. First, 0.2 mL of 0.05 M silver nitrate (AgNO_3_) was mixed in 96.56 mL of deionized water. Next, the mixture was vigorously stirred (900 rpm) at room temperature. Afterward, 2.0 mL of 75 mM trisodium citrate was added into the mix and then 0.24 mL of 30% hydrogen peroxide (H_2_O_2_) was added. Then, 0.1 M sodium borohydride (NaBH_4_) as the reducing agent was injected quickly into this mixture. After undergoing vigorous stirring for 5 min, the clear and colorless solution turned into a dark-blue solution, indicating the formation of a AgNP triangle. Finally, the solution was centrifuged at 6000 rpm for 30 min.

Preparation of a AgNP LSPR glass slide via a drop-cast method: The drop-cast method was prepared for the 1 cm × 1 cm glass substrates. We began this method by washing the glass with liquid detergent and deionized water and then immersing it in a piranha solution (concentrated sulfuric acid and 30% hydrogen peroxide at a ratio of 3:1 v/v) for 1 h at room temperature to eliminate any organic waste on the glass surface. Following this, the substrate was washed in acetone and then ultrasonically treated in ethanol for 15 min. Then, the hydroxylated surface on the clean silicon was submerged in 5% (3-aminopropyl) triethoxysilane (APTES) in ethanol for 1 h. The salinized glass substrate was then thoroughly washed with a large amount of ethanol to remove the unbound molecules. This APTES film was entirely dried on a hot plate at 30 °C. The pellet produced at the bottom of the centrifuge tube was extracted, diluted with the deionized water, and deposited on the glass surface.

**Figure 2:**
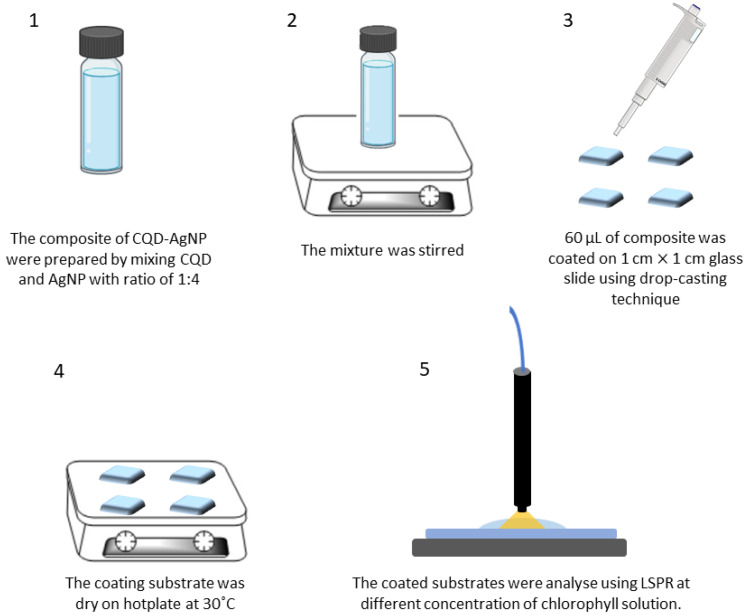
Experimental procedure for the preparation of a substrate coated with AgNPs and a AgNP–CQD composite.

Meanwhile, the composite of AgNP–CQD was prepared by mixing CQDs and AgNPs in a ratio of 1:4. Then the mixture was stirred, and 60 µL of the composite was coated on a 1 cm × 1 cm glass slide using the drop-casting technique and dried on the hot plate at 30 °C. The coated substrates were analyzed using LSPR. Figure 2 depicts the procedure of preparation of glass slides using the composite mixture.

**Figure 3:**
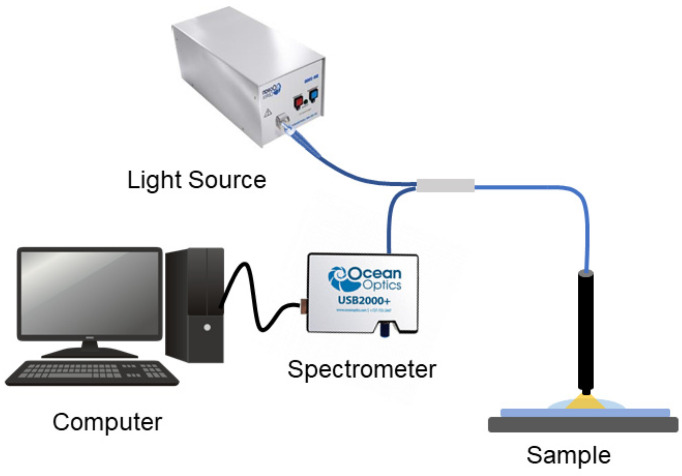
Experimental setup for the detection of chlorophyll using LSPR.

Detection of chlorophyll: The system comprises three components: a light source (HR4000CG-UV-NIR, Ocean Optics Inc., Dunedin, FL, USA), a 400–1000 nm DH-2000-BAL (Ocean Optics) spectrometer, and a reflection probe with a numerical aperture of 0.22 between the source and the detector. As illustrated in Figure 3, the probe was fixed to an optimum height above the sample. Reflectivity spectra of bare AgNPs and the AgNP–CQD composite with different chlorophyll concentrations (0.1–10 ppm) were collected across a 300–850 nm wavelength range, with a few minutes of spectral capture time.

Characterization process: A Fourier-transform infrared spectrum (PerkinElmer, Waltham, Massachusetts, USA) was used to analyze the functional groups on the CQD surface within the scan range of 4000–400 cm^−1^. An X-ray photoelectron spectrum (XPS) was conducted on Axis Ultra DLD. Kratos/Shimadzu uses Al/Kα as the supply to determine the composition and chemical bonding configuration. The particle size and morphology of synthesized CQD were characterized by a transmission electron microscope using Talos L120C. The absorption spectrum of CQD was obtained using a UV–vis spectrophotometer (Perkin Elmer- Lambda 950, Waltham, Massachusetts, USA) and photoluminescence (PL) emission studies recorded on a photoluminescence spectrometer (FLS920, Edinburgh Instrument, Livingston, UK). The normalized fluorescence spectrum was generated by dividing the highest intensity value by each fluorescence intensity point by point. Finally, the fluorescence lifetime of CQD without or with the addition of chlorophyll was analyzed with a time-correlated single-photon counting (TCSPC) device and anisotropy analysis. 

## 3. Results

### 3.1. Characterization of As-Synthesized CQDs and AgNPs

**Figure 4:**
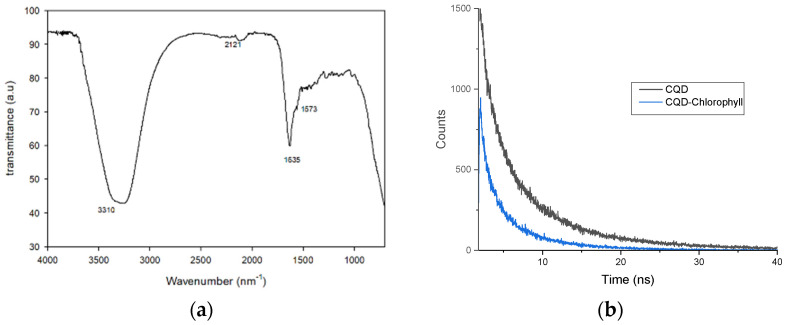
(**a**) Infrared spectra of a bare CQD. (**b**) Decay curve of a CQD and a CQD-chlorophyll.

CQDs were prepared in one step of the hydrothermal technique using citric acid and ethylenediamine as carbon and nitrogen source precursors. The functional groups and composition structure of the CQDs were investigated through FTIR measurement and XPS analysis, respectively. As illustrated in FTIR spectra (Figure 4), the peaks at 1635 cm^−1^, 1573 cm^−1^, and 2121 cm^−1^ are ascribed to C–O, N–C=O, and C≡C stretching vibrations, respectively [24]. The broad and strong peak at 3000–3500 cm^−1^ corresponds to the stretching mode of the OH/NH_2_ group [17]. The presence of OH and NH indicates the existence of functional groups that influence the stability of CQDs in aqueous states [25]. The average fluorescence lifetime (τ_ave_) of CQDs was determined as 0.0346 ns, shown in Figure 4b. The fluorescence lifetime is significantly reduced to 0.0142 ns after adding chlorophyll. The reduced fluorescence lifetime demonstrated that the fluorescence quenching induced by chlorophyll was probably influenced by electrostatic interaction in the CQD and chlorophyll system [18].

**Figure 5:**
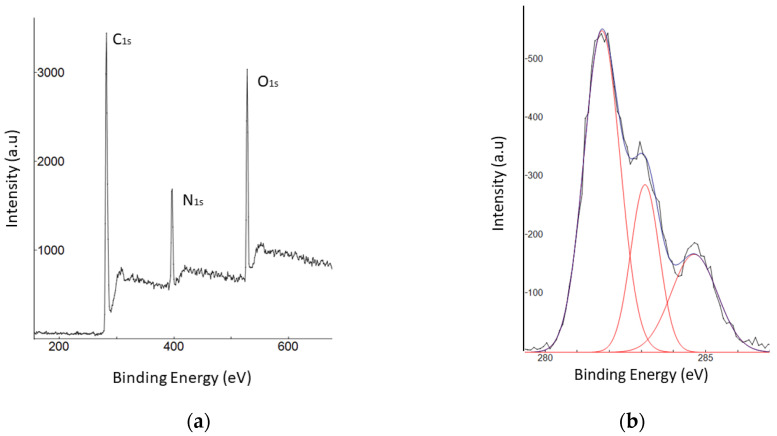

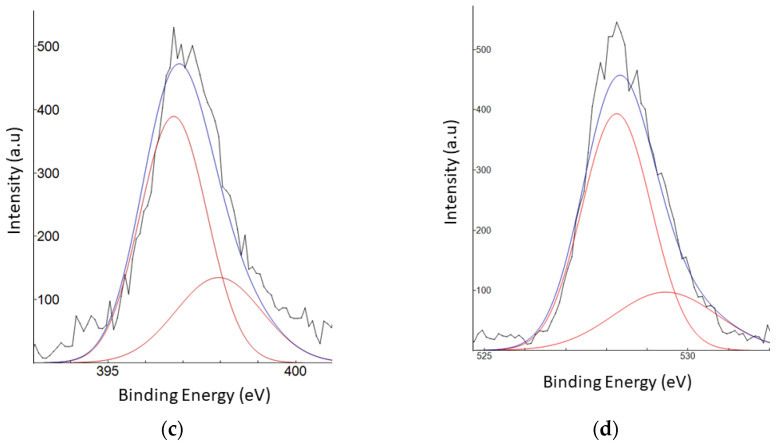
XPS spectrum (**a**) and high-resolution XPS spectrum of (**b**) C1s, (**c**) N1s, and (**d**) O1s for CDs.

XPS measurements were carried out to investigate the details of CQD composition. As shown in Figure 5a, the survey spectrum discloses three prominent peaks: C1s at 284 eV, N1s at 396 eV, and O1s at 528 eV. Thus, the CQD is composed of C, N, and O components. The XPS spectrum (Figure 5b) was calibrated to the position of C1s. The C1s spectrum decomposed into three peaks, at 281.7, 283.5, and 284.6 eV, which can be assigned to C=O, C–C, and C=C, respectively [26,27]. The N1s spectra (Figure 5c) reveal N atoms’ presence in H-N-C_2_ at 397.1 eV and oxidation state of N^2−^ at 399.5 eV. The O1s spectrum in Figure 5d demonstrates two prominent bands, at 528.9 and 529.6 eV, representing the form of the binding energy of lattice oxygen and C=O, respectively [28]. All the XPS results correlate with the FTIR results. The results from XPS and FTIR have been validated to confirm that CQDs consist of several surface functional groups (hydroxyl, amide, amino, and carboxyl/carbonyl compounds) [25].

**Figure 6:**
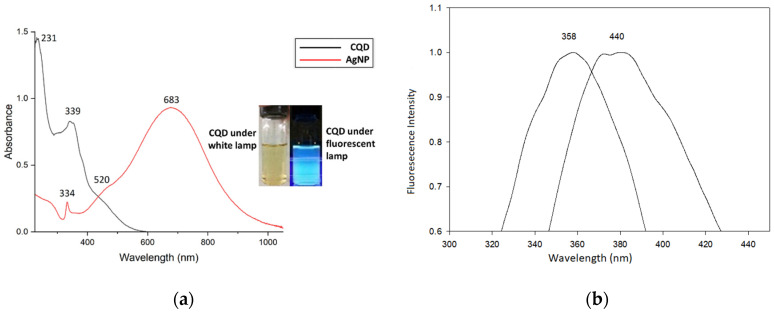
(**a**) Absorption peak of CQDs (black line) and AgNPs (red line) and (**b**) excitation (358 nm) and emission (440 nm) of CQDs.

In Figure 6a, the optical properties of the prepared CQDs have absorption bands at 231 and 339 nm (black line). The first characteristic absorption peak, at 231 nm, belongs to the C=C bond p–p* transition. The peak at 339 nm stands for the n–p* transition, which depends on the emission trap formation and excitation surface states that facilitate fluorescence enhancement [29]. Meanwhile, the AgNP shows three absorption peaks (red line). The absorption bands at 683, 520, and 334 nm are assigned to in-plane dipole, in-plane quadrupole, and out-of-plane quadrupole plasmon resonance of the AgNP, respectively [13]. Under white light, the CQD solution appears bright yellow in the right inset of Figure 6a. The CQDs emitted a bright-blue light when excited under a 365 nm wavelength. In the fluorescence spectra (Figure 6b), the optimum excitation and emission wavelengths for CQDs are 358 and 440 nm, respectively.

**Figure 7:**
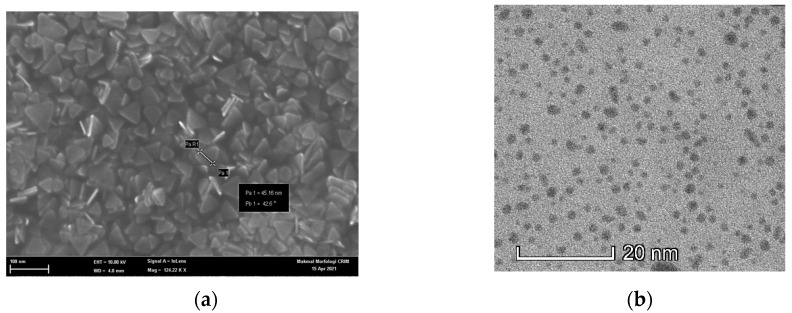
Morphology of sensing material with the analyte (**a**) FESEM of triangular AgNPs and (**b**) TEM of CQDs.

The morphology of the prepared CQDs and AgNPs were analyzed by FESEM and TEM imaging. The FESEM image demonstrates that the Ag nanoplates were synthesized with a high yield of triangular shapes (Figure 7a). The mean side length of the triangular AgNP is 50 nm, calculated using Image J software. The results show that the side length of 50 nm is detected on the surface, which is supported by the zeta sizer analysis. The particle size of AgNPs was found on average to be 16.73 nm with good distribution. TEM imaging shows that CQDs are uniform in size (below 10 nm), quasi-spherical shaped, and with high monodispersity (Figure 7b). They were freely dispersed in water due to the small dimensions and the functional groups of carboxyl and amino groups originating from the citric acid and ethylenediamine during the synthesis process.

### 3.2. Sensing Performance

LSPR sensors use AgNPs and AgNP–CQD as detection materials to detect chlorophyll. Therefore, the capabilities of these two materials were compared. In this research, we used 90% acetone as our baseline for both AgNPs and AgNP–CQD. Thus, 90% of acetone and five different chlorophyll concentrations, ranging from 0.2 to 10.00 ppm, were prepared. The baseline wavelength of reflectance was recorded when the glass substrate was in contact with acetone, and a new reflection peak emerged as the analyte concentration changed. The change between the wavelength (Δλ) and the chlorophyll concentration was calculated as the sensitivity of the LSPR sensor, using the first reflectance peak as a reference and comparing it to the new reflectance peak [30].

**Figure 8:**
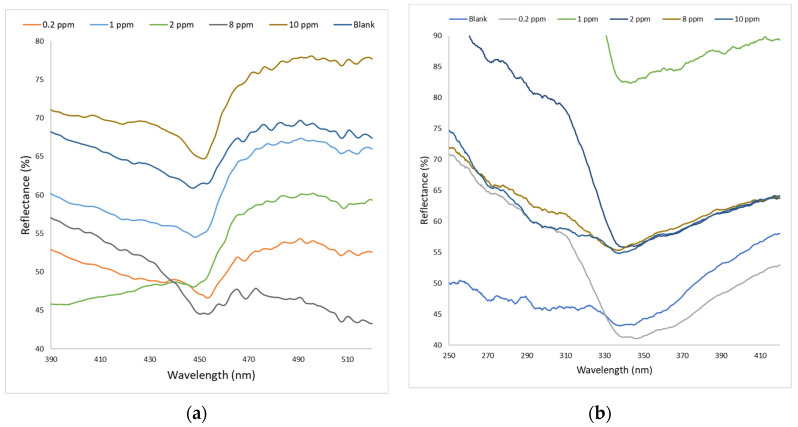
LSPR spectra for the detection of chlorophyll at different concentrations using (**a**) AgNPs and (**b**) a AgNP–CQD composite.

Figure 8a depicts the spectrum of AgNPs showing a narrow and strong peak when disclosed to diverse concentrations of chlorophyll. The reflection peaks at 453.35, 448.37, 447.59, 453.61, and 451.52 nm are shifted from the reference peak (440.24 nm) for 0.20, 1.00, 2.00, 8.00, and 10.00 ppm, respectively. Different amounts of chlorophyll lead to the spectrum’s wavelength shifts, resulting from the interaction between chlorophyll and AgNPs. It is plausible to assume that the wavelength shift is caused by the electron transfer mechanism from excited chlorophyll to AgNPs [31]. In addition, some reports have demonstrated that porphyrin derivatives could undergo electron transfer with AgNPs [31,32] and the intensity of chlorophyll emission can be suppressed by gold nanoparticles caused by the photoinduced electron transfer [33].

At the same time, Figure 8b illustrates the reflectance spectrum of AgNP–CQD when evaluated for chlorophyll detection. As shown in Figure 8b, a broad and low-intensity peak was observed when AgNP–CQD was exposed to various chlorophyll concentrations. The reflectance peak trend in Figure 8b was measured to be shifted when AgNP–CQD came in contact with lower concentrations of chlorophyll. The initial peak is 345.31 nm, where minimum reflectance occurred for 90% acetone. We discovered that the spectral peak of the reflectance is positively correlated with the increase in chlorophyll concentration. Thus, the measured Δλ values for 0.20, 1.00, 2.00, 8.00, and 10.00 ppm of chlorophyll are 0.79, 2.13, 4.25, 7.69, and 9.28 nm, respectively. The observed wavelength shift was due to electrostatic interaction, as confirmed by the average fluorescence lifetime in Figure 4b. Many methods have found that when the interacting substances are oppositely charged, electrostatic interaction can increase the rate of mutual diffusion of the reactants because they are close to each other. Therefore, the quencher rate constant of a pair of oppositely charged emitter–quencher pairs may exceed the typically limited diffusion rate constant of neutral transmitter emitter–quencher pairs.

**Figure 9:**
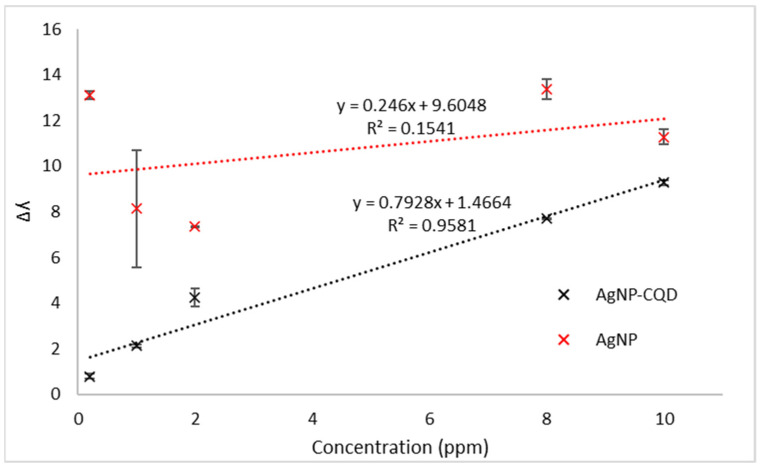
The LSPR calibration curve of bare AgNPs (red) and a AgNP–CQD composite (black) detects chlorophyll ranging from 0.2 to 10 ppm concentration.

Figure 9 shows the calibration plot of AgNPs and AgNP–CQD for three repeated experiments with chlorophyll concentrations in the range of 0.20 to 10.0 ppm. The graph demonstrated that the Δλ increases proportionally with chlorophyll concentrations for the AgNPs and the AgNP–CQD composite. Figure 9 demonstrates a graph for comparing the linear regression of AgNPs and AgNP–CQD. When both materials are used to detect different chlorophyll concentrations, AgNP–CQD shows a high correlation coefficient (R^2^ = 0.9581) compared to AgNPs (R^2^ = 0.1541). The slope of the graph determined the sensor’s sensitivity. The detection accuracy for the AgNP–CQD composite is higher than that of AgNPs, showing a better performance for the LSPR sensor. Remarkably, the AgNP–CQD sensor sensitivity was determined to be 0.80 nm ppm^−1^, while the sensitivity of AgNPs was estimated at 0.25 nm ppm^−1^. The functional groups bonded to the surface of carbon quantum dots resulted in higher sensitivity for AgNP–CQD, as demonstrated by the FTIR spectra in Figure 4a and XPS analysis in Figure 5. These CQDs can provide more binding sites for chlorophyll. Hence higher sensitivity was obtained for AgNP–CQD compared to AgNPs. Based on this finding, it was hypothesized that the inclusion of CQDs significantly increased the sensitivity of the LSPR sensor. It has previously been claimed that portable fluorometers capable of detecting chlorophyll-a in situ are now accessible [34]. However, Hamdhani et al. suggested that the efficiency of the fluorometer became lower when the chlorophyll-a concentrations were greater than 25 ppb and turbidity levels were less than 50 NTU. However, this work obtained an excellent sensitivity with the detection range (0.2–10 ppm), primarily due to the addition of CQDs as the sensor layer of chlorophyll detection. Based on these findings (Table 1), it was demonstrated that AgNP–CQD outperforms AgNPs as a sensing material in terms of chlorophyll detection. The results of this study indicate that nano-based sensors have the potential to operate in the solid phase, for example, the integration of AgNP–CQD with optical microfiber sensor technology [35]. An optical fiber LSPR sensor has the advantages of small size and simple produced process. CQDs and silver nanoparticles as coating materials on the fiber can increase the sensitivity of the LSPR sensor because the shape and material of the nanoparticles significantly influence the sensitivity [36,37].

## 4. Conclusions

This research developed AgNPs and a AgNP–CQD composite as the active layer for LSPR-based chlorophyll detection. A AgNP–CQD composite shows better linearity (R^2^ = 0.9581) and high sensitivity (0.80 nm ppm^−1^) as a sensing material for chlorophyll detection compared to AgNPs (R^2^ = 0.1541, 0.25 nm ppm^−1^). The LOD of chlorophyll was lowered by more than an order of magnitude with AgNP–CQD (4.71 ppm) compared to AgNPs alone (52.76 ppm). Furthermore, the AgNP–CQD composite shows high sensitivity due to the functional groups attached to the CQDs of its structure, which are NH_2_, OH, and COOH, as proven by FTIR and XPS analysis. The inclusion of functional groups increases the active area for chlorophyll interaction with the AgNP–CQD surface. These results demonstrate that the AgNP–CQD composite is a remarkable sensing material for detecting chlorophyll compared to AgNPs. Our findings suggest that the proposed AgNP–CQD LSPR platform can be used to detect chlorophyll with high sensitivity and low LOD. Furthermore, considering the potential of optical fiber application, this simple and efficient detection technique could be applied for chlorophyll monitoring of the ocean population.

## Figures and Tables

**Table 1:** The performance and correlation coefficient R^2^ of AgNPs and a AgNP–CQD composite for chlorophyll detection.

Compound	R^2^	Sensitivity	Range (ppm)	LOD (ppm)	LOQ (ppm)
AgNP	0.1541	0.25	0.2–10.0	52.76	175.87
AgNP–CQD	0.9581	0.80	0.2–10.0	4.71	15.70

## Data Availability

Not applicable.
